# Protective effect of *in ovo* treatment with the chicken cathelicidin analog D-CATH-2 against avian pathogenic *E. coli*

**DOI:** 10.1038/srep26622

**Published:** 2016-05-27

**Authors:** Tryntsje Cuperus, Albert van Dijk, Mieke G. R. Matthijs, Edwin J. A. Veldhuizen, Henk P. Haagsman

**Affiliations:** 1 Division of Molecular Host Defence, Department of Infectious Diseases & Immunology, Faculty of Veterinary Medicine, Utrecht University, Utrecht, The Netherlands; 2 Division of Poultry Health, Department of Farm Animal Health, Faculty of Veterinary Medicine, Utrecht University, Utrecht, The Netherlands

## Abstract

Increasing antibiotic resistance and ever stricter control on antibiotic use are a driving force to develop alternatives to antibiotics. One such strategy is the use of multifunctional Host Defense Peptides. Here we examined the protective effect of prophylactic treatment with the D analog of chicken cathelicidin-2 (D-CATH-2) against a respiratory *E. coli* infection. Chickens were treated with D-CATH-2 *in ovo* at day 18 of embryonic development or intramuscularly at days 1 and 4 after hatch. At 7 days of age, birds were challenged intratracheally with avian pathogenic *E. coli*. Protection was evaluated by recording mortality, morbidity (Mean Lesion Score) and bacterial swabs of air sacs at 7 days post-infection. *In ovo* D-CATH-2 treatment significantly reduced morbidity (63%) and respiratory bacterial load (>90%), while intramuscular treatment was less effective. D-CATH-2 increased the percentage of peripheral blood lymphocytes and heterophils by both administration routes. *E. coli* specific IgM levels were lower in *in ovo* treated animals compared to intramuscular D-CATH-2 treatment. In short, *in ovo* treatment with the Host Defense Peptide derived D-CATH-2 can partially protect chickens from *E. coli* infection, making this peptide an interesting starting point to develop alternatives to antibiotics for use in the poultry sector.

Host Defense Peptides (HDPs) are important effector molecules of the innate immune system. These peptides have been found in a wide range of organisms from plants to insects and mammals. One of the main classes of HDPs comprises the cathelicidins; short, cationic and amphipathic peptides with a highly diverse sequence but conserved structure. While in some species this class of HDPs is represented by a single molecule (LL-37 in humans, CRAMP in mice), a wider repertoire of cathelicidins is found in other animals with the pig genome encoding for eleven of these peptides^1^. The chicken has four cathelicidins (CATH-1-3 and –B1). Cathelicidins were initially described as antimicrobial peptides, able to efficiently kill a variety of bacteria^2^. In the last decade, their immunomodulatory functions increasingly have been identified. Cathelicidins are now known to be involved in among others chemotaxis, differentiation of leukocytes and modulation of cytokine responses, making these peptides truly multifunctional^3^. Most research efforts are focused on cathelicidins from mammals, but non-mammalian peptides such as avian cathelicidins are increasingly investigated and their functions seem to overlap with the mammalian counterparts^4^.

Infections with avian pathogenic *E. coli* (APEC) are among the most important causes of mortality and morbidity for poultry, causing large economic losses in the industry^5^. APEC infections of respiratory origin cause lesions in the respiratory tract, frequently followed by a generalized infection of the internal organs and septicemia. These infections mostly occur in immunocompromised birds, for example in combination with viral infections or in very young animals^6^. The treatment of APEC infections currently relies heavily on antibiotics, but the high antibiotic resistance in APEC strains makes this more and more problematic^7^.

Antibiotic resistance is a large and growing problem in both veterinary and human medicine. This has caused an intense search for alternative means to prevent and fight infections. Cathelicidins or derivatives thereof are investigated in areas as diverse as periodontology^8^, skin infections^9^, biofilm related problems^10^ and bovine mastitis^11^ and show promise as alternatives to antibiotics with the added benefit of inducing little to no resistance^12^.

Chicken cathelicidin-2 (CATH-2) has previously shown multiple immunomodulatory effects including inhibition of LPS-induced effects and induction of chemokines^13^,^14^.

In this article we evaluate the prophylactic anti-infective efficacy of the D-amino acid analog of CATH-2 in young broiler chickens infected with avian pathogenic *E. coli*. The D-analog was chosen because of the higher stability of D-amino acid based peptides in biological fluids^15^. D-CATH-2 was administered either by injection into the egg (*in ovo*) three days before hatch or intramuscularly (i.m.) at day of hatch and day 4 of age. At 7 days of age, chickens were intratracheally inoculated with *E. coli* and animals were monitored for a week. Treatment with D-CATH-2 was shown to partially protect chickens against APEC infection with *in ovo* injection of the peptide showing a greater efficacy compared to i.m. administration.

## Results

### CATH-2 derived peptides localize to gastro-intestinal and respiratory tract after *in ovo* injection

**Figure 1:**
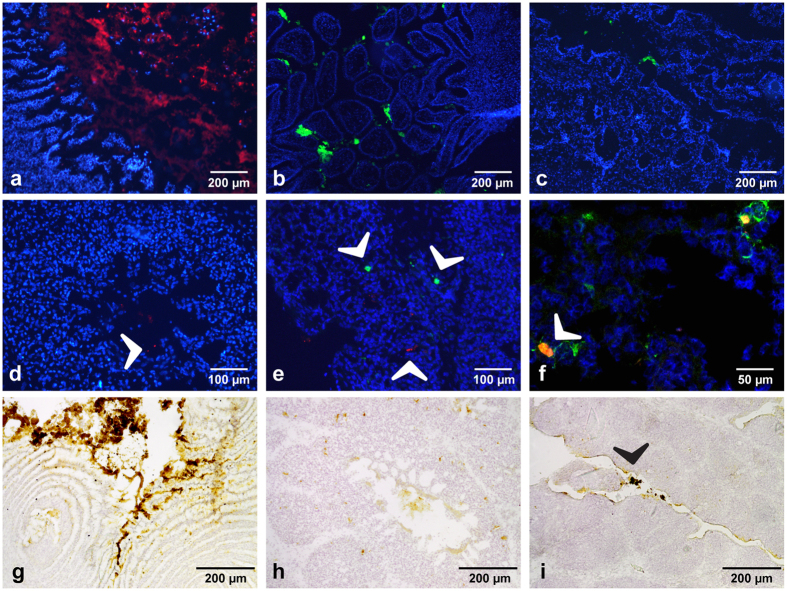
Localization of peptides 24 hours after *in ovo* injection. Peptides were labeled with fluorescein isothiocyanate (FITC) or carboxytetramethylrhodamine (TAMRA). Using fluorescent microscopy, peptides were found in great abundance in the upper GI tract: (**a**) TAMRA-L-CATH-2 in proventriculus, (**b**) FITC-D-CATH-2 in duodenum), and infrequently also in the more distal intestine: (**c**) FITC-D-CATH-2 in cecum Peptides were found spread throughout the lung: (**d**) TAMRA-D-CATH-2, and incidentally co-localized with macrophages ((**e**,**f**) KUL-01 green, TAMRA-L-CATH-2 red). Immunohistochemistry confirmed these results: (**g**) TAMRA-L-CATH-2 in proventriculus, (**h**) TAMRA-L-CATH-2 in lung. Small lumps of FITC-D-CATH-2 were found in the lumen of the bursa of Fabricius (**i**).

In the embryonated chicken egg, multiple fluid-filled compartments are present: the amniotic fluid, the allantoic fluid and the yolk sac. In this study, *in ovo* injection was aimed for the amniotic fluid to mimic the commercially used *in ovo* vaccination technique. To discover the distribution of *in ovo* injected peptides, fluorescently labeled CATH-2 analogs were injected into 18-day embryonated eggs. At 24 hours post injection, peptides, both FITC-D-CATH-2 and TAMRA-L-CATH-2, were found in the gastro-intestinal (GI) tract, the respiratory tract and on the skin of the embryos. Fluorescence was not detected in the tissues of embryos injected with buffer only. The observation of a thin layer of peptide covering the skin of the animals indicated peptide had indeed been administered into the amniotic fluid, which surrounds the embryo. For both peptides, a large amount was seen in the glandular (proventriculus, Fig. 1a) and muscular (gizzard) stomach. Labeled peptides were even visible macroscopically by the color of the stomach contents. In the duodenum, peptide could clearly be seen surrounding the villi (Fig. 1b). Observations of peptides became more infrequent in distal parts of the GI tract. However, small fluorescent lumps were observed in the lumen of ileum, jejunum, cecum (Fig. 1c) and colon. Neither of the peptides could be detected inside enterocytes or other cells of the intestinal wall. In the respiratory tract, peptides were found in the lumen of trachea and bronchi and spread throughout the entire lung, as far as the air capillaries (Fig. 1d), the smallest anatomical unit of the avian lung. As in the GI tract, peptide localization was mostly extracellular. Yet, staining of lung sections with a monocyte/macrophage (α-KUL01) antibody indicated that uptake of peptides by mononuclear phagocytes incidentally occurred (Fig. 1e,f). The location of peptides found with fluorescence microscopy was confirmed and elucidated by analysis of tissue sections with immunohistochemistry (Fig. 1g,h). Using this technique, it was possible to show presence of FITC-D-CATH-2 in the lumen of the bursa of Fabricius and uptake by the bursal epithelium (Fig. 1i). In the bursa TAMRA-L-CATH-2 could not be detected, but overall localization of the L- and D-version of the peptide was similar.

### Protection against *E. coli* induced mortality and morbidity after D-CATH-2 treatment

**Figure 2:**
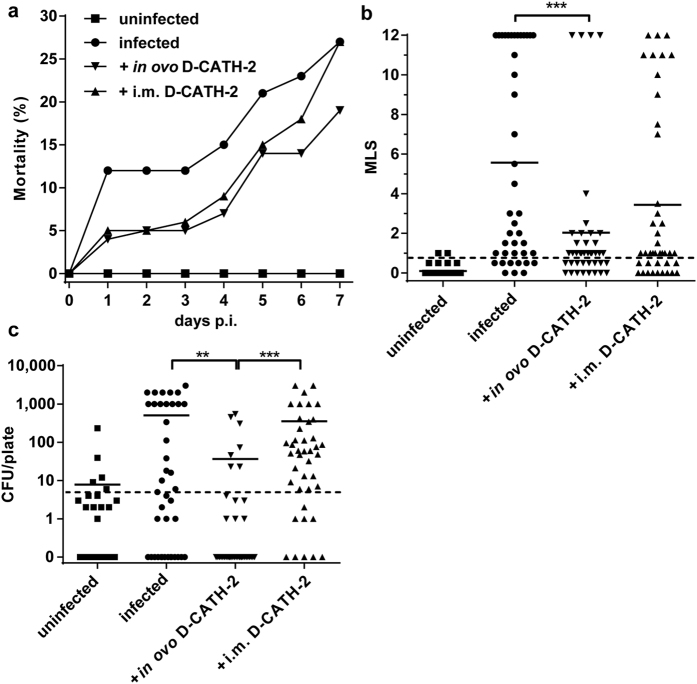
*E. coli* related mortality (a), morbidity (b) and bacterial counts in air sacs (c) at 7 days post challenge. Chickens were treated *in ovo* with 1 mg/kg bodyweight D-CATH-2 or i.m. at day of hatch and day 4 with 20 and 2.5 mg/kg bodyweight D-CATH-2. At 7 days post hatch, chickens were inoculated intratracheally with 10^7^ CFU of *E. coli* 506. Morbidity is represented as Mean Lesion Score, the sum of gross pathology scored in air sacs, liver and pericardium (**b**). Dotted line indicates MLS = 1, above which animals are considered colibacillosis positive (Table 1). Air sac swabs were plated on MacConkey/Sheep Blood agar plates and colonies were counted after overnight incubation (**c**). Dotted line indicates CFU = 5, above which animals are considered *E. coli* positive (Table 2). Mortality data were analyzed by the Kaplan-Meier method, morbidity results and *E. coli* counts were analyzed by one-way ANOVA with a Games-Howell post-hoc test for non-equal variances. **p < 0.01, ***p < 0.001.

To determine the effect of D-CATH-2 administration on the outcome of a respiratory *E. coli* challenge, mortality was recorded for 7 days after infection and morbidity was evaluated in surviving birds at 7 days post infection (p.i.). In the uninfected animals, no mortality was seen. In the untreated challenged group, 27% of the chickens died within 7 days p.i (Fig. 2a). Mortality was not significantly reduced by *in ovo* or intramuscular D-CATH-2 treatment, though at 7 days p.i., 30% less animals had died in the *in ovo* D-CATH-2 treated group (p = 0.155). *E. coli* associated morbidity was analyzed by assigning a Mean Lesion Score (MLS). Chickens treated *in ovo* with D-CATH-2 showed a drastically decreased morbidity compared to the untreated infected animals (p = 0.001, Fig. 2b). The group of chickens treated i.m. with D-CATH-2 showed a trend towards a lower MLS (p = 0.055). In addition to decreased severity of lesions, both D-CATH-2 treatments significantly (p = 0.004 and p = 0.046 respectively) reduced the number of chickens with colibacillosis lesions (MLS > 1, Table 1).

### Decreased *E. coli* colonization of air sacs after *in ovo* D-CATH-2 administration

The observed reduction of mortality and morbidity by D-CATH-2 treatment is expected to correlate with decreased bacterial colonization. Therefore, air sac swabs and blood samples were analyzed for the presence of *E. coli*. Thoracic air sacs were heavily colonized by *E. coli* at 7 days p.i. in untreated infected birds. Air sac colonization was greatly reduced for *in ovo* peptide treated birds, but not for animals treated i.m. with D-CATH-2 (p = 0.002 and p = 0.307 respectively, Fig. 2c). Also, the number of animals with a positive air sac swab (CFU > 5) was significantly lower in the *in ovo* D-CATH-2 treated chickens compared to untreated infected animals (p = 0.004, Table 2). In contrast, the number of animals with *E. coli* positive air sacs was significantly higher in the group treated i.m. with D-CATH-2 (p = 0.011). In peripheral blood samples, *E. coli* numbers were generally low at 7 days post infection (Supplementary Fig. S1). *E. coli* numbers in blood at 7 days p.i. showed a trend towards decrease (p = 0.094) in the *in ovo* D-CATH-2 treated group. Similar to the results for air sac colonization, the number of chickens with *E. coli* positive blood cultures was significantly lower in the group treated *in ovo* with D-CATH-2 compared to the untreated infected animals (p = 0.014, Table 2).

### Increased leukocyte and heterophil numbers in blood of D-CATH-2 treated animals

**Figure 3:**
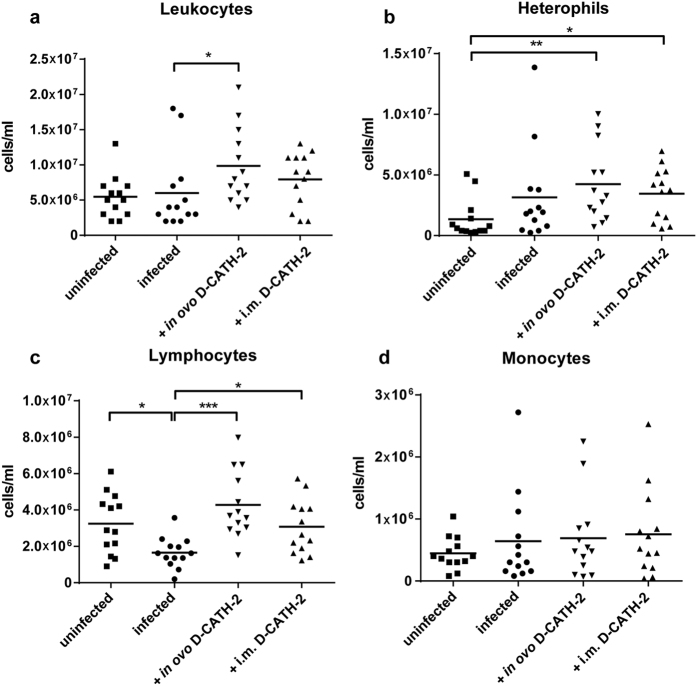
Leukocyte counts in peripheral blood at 2 days post *E. coli* challenge based on blood smear analysis. (**a**) leukocytes, (**b**) heterophils, (**c**) lymphocytes, (**d**) Monocytes. Data were analyzed by one-way ANOVA with a Tukey post-hoc test. *p < 0.05, **p < 0.01, ***p < 0.001.

Peripheral blood smears were prepared to examine the influence of *E. coli* infection and D-CATH-2 treatment on total leukocyte counts and leukocyte populations. *E. coli* infection did not affect the total number of leukocytes or heterophils but significantly decreased lymphocyte counts (p = 0.016, Fig. 3). Animals treated *in ovo* with D-CATH-2 showed an increased total number of leukocytes (p = 0.047) and heterophils (p = 0.004) and prevented *E. coli* induced lymphopenia (p = 0.0002). To a lesser extent intramuscular D-CATH-2 treatment exhibited a similar effect on the heterophil (p = 0.015) and lymphocyte populations (p = 0.021), but did not augment the number of total leukocytes. Monocyte numbers were not significantly affected by infection or treatment (Fig. 3d).

### Antibody levels after *E. coli* challenge

**Figure 4:**
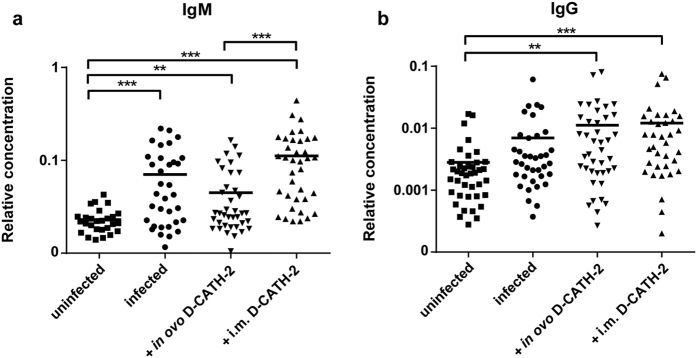
*E. coli* specific antibody levels in serum at 7 days post challenge. Antibodies were measured using an ELISA. (**a**) IgM, (**b**) IgG. Relative concentrations were calculated based on a standard curve of pooled serum samples. Data were analyzed by one-way ANOVA using a Games-Howell post-hoc test for non-equal variances for IgM results and a Tukey post-hoc test for IgG results. **p < 0.01, ***p < 0.001.

As antibodies were previously shown to be protective in avian *E. coli* infection^16^,^17^, *E. coli* specific antibody levels were measured in serum at 7 days post challenge to look at the effect of D-CATH-2 treatment on humoral immunity. Infection with *E. coli* led to increased IgM levels (p = 0.0004), but did not significantly augment IgG production (Fig. 4). In contrast, *in ovo* and intramuscular D-CATH-2 treatments significantly increased both IgM (p = 0.007) and IgG levels (p = 0.001) at 7 days p.i. compared to uninfected animals. The level of IgM antibodies seemingly decreased in animals treated *in ovo* with D-CATH-2 compared to the untreated infected group (p = 0.248). Significantly higher levels of IgM antibodies were seen in the i.m. treated group compared to the *in ovo* treated group (p = 0.00006, Fig. 4a).

### Histological parameters in the lung after *E. coli* challenge

**Figure 5:**
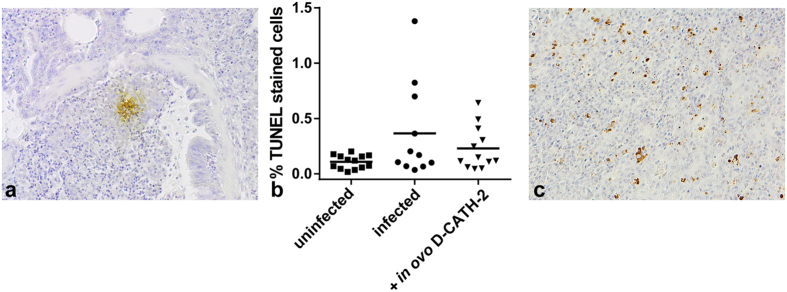
Histological parameters of lungs at 2 days post *E. coli* challenge. (**a**) Representative example of immunohistochemical staining of *E. coli* in lung of an infected animal (200×). (**b**) Percentage of apoptotic cells in lungs as determined by TUNEL assay. Data were analyzed by one-way ANOVA using a Tukey post-hoc test. (**c**) Representative example of TUNEL stain in lung of an infected animal (400×).

Pathology of the *E. coli* infection was evaluated in the lungs of the chickens at 2 days p.i. Presence of *E. coli* in the lungs was determined by immunohistochemistry, the number of apoptotic cells was analyzed using the TUNEL assay. *E. coli* staining demonstrated that at 2 days post infection bacteria were present in the lungs of approximately 50% of the infected animals, independent of treatment. Bacteria were mostly found in areas of the lung with clear signs of histopathology such as congestion and leukocyte influx (Fig. 5a). The number of apoptotic cells was low and not significantly different between infected and non-infected animals (p = 0.133), though in a few infected animals areas with increased apoptotic cell numbers were found (Fig. 5b). Similar to *E. coli* localization, large numbers of apoptotic cells were only found in areas with clear histopathology (Fig. 5c).

### Immune gene expression in lung and spleen after *E. coli* challenge

**Figure 6:**
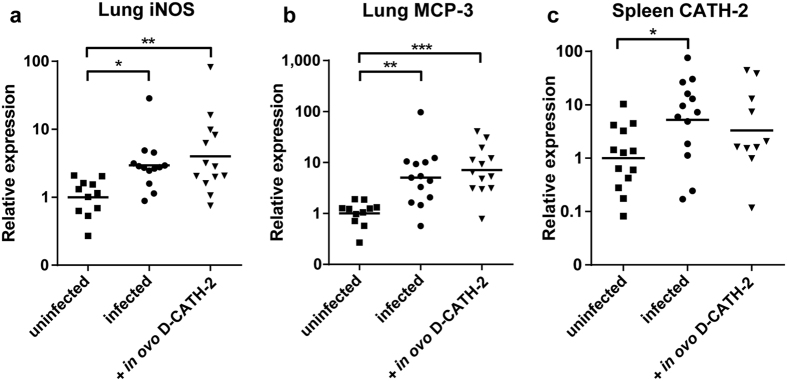
Immune related gene expression measured at 2 days post *E. coli* challenge by qPCR. Expression was calculated relative to the uninfected group. Geometric means are displayed. (**a**) iNOS in lung, (**b**) MCP-3 in lung, (**c**) CATH-2 in spleen. Data were analyzed by one-way ANOVA with a Tukey post-hoc test. *p < 0.05, **p < 0.01, ***p < 0.001.

Expression of innate immune related genes previously shown to be important in avian *E. coli* infections or in effects of HDPs was measured by qPCR in lung and spleen of chickens at 2 days post *E. coli* challenge^18^,^19^. Expression in general showed large inter-animal variation and little difference between treatment groups. *E. coli* infection significantly increased expression of inducible nitric oxid synthase (iNOS, p = 0.027) and the chemokine MCP-3 (p = 0.002) in the lung and expression of endogenous CATH-2 (p = 0.044) in the spleen (Fig. 6). No difference was apparent between non-treated and D-CATH-2 treated animals. Expression of several other innate immune related genes such as the β-defensin AvBD9 and chemokines RANTES and IL-8 in the lung and IL-1β and IL-6 in the spleen were not significantly altered by infection or D-CATH-2 treatment (Supplementary Figs S2 and S3).

## Discussion

In the present work we report that an analog of the chicken Host Defense Peptide CATH-2 was able to protect young chickens against *E. coli* induced mortality and morbidity. These results show that peptides based on D-CATH-2 and HDPs in general have the potential to be developed as new anti-infective drugs.

In this study, we used *in ovo* injection of D-CATH-2. *In ovo* administration of vaccines is common practice in hatcheries around the world to protect chickens against viral infections such as Marek’s disease, Newcastle disease and infectious bursal disease^20^. The technique has many advantages compared to post-hatch vaccination including earlier protection, reduced stress of the chicks and lower costs of administration. These advantages have made researchers look at the opportunities of administrating other substances beyond vaccines to benefit poultry health. *In ovo* trials have been conducted among others with (micro)nutrients, interleukins and CpG-ODN^21^,^22^,^23^. *In ovo* injection at the 18-day embryonated stage is aimed towards deposition in the amniotic fluid surrounding the embryo which is taken up by the developing chick towards the end of incubation^24^. Because the characteristics of injected substances influence their ultimate anatomical location^25^, we determined the localization of CATH-2 analogs after *in ovo* injection. Both CATH-2 analogs were detected in the gastro-intestinal and respiratory tract of the embryos. These results clearly show how the peptides are imbibed with the amniotic fluid by the embryo and prove *in ovo* injected peptides can reach and influence these two anatomical systems. Localization was largely similar for both CATH-2 analogs, the L-peptide and the protease resistant D form. This indicates that possible breakdown of the L-analog does not prevent this peptide from reaching tissues to be affected. Cellular uptake by the epithelial cells lining the GI and respiratory tract of L- or D-CATH-2 could not be detected. However, uptake was incidentally shown for KUL-01 positive cells in the lung and epithelial cells in the bursa. Uptake of these CATH-2 analogs by chicken macrophages was previously shown *in vitro*^26^.

In this study we used a well-described intratracheal model which mimics the natural infection route of *E. coli* in chickens^27^. We were able to show that D-CATH-2 is able to partially protect young chicks from *E. coli* related morbidity with *in ovo* administration being more efficient compared to i.m. administration after hatch. Previously, we showed that CATH-2 analogs efficiently kill *E. coli in vitro*^14^,^28^. In the experiment described here, *E. coli* numbers were decreased in the air sacs of the D-CATH-2 treated groups. However, direct antibacterial action is very unlikely in this model. With the small amount of peptide administered in the eggs (0.022 mg/egg) and the rapid increase of bodyweight of the chick (over 8 times between an 18 day old embryo and a 7 day old chick) the concentration of peptide still present in the organs would be extremely low at the moment of *E. coli* challenge. In our opinion a more likely hypothesis is that D-CATH-2 is able to modulate the immune system, resulting in a better protection against future infections. Recent studies in our group have shown strong immunomodulatory activity of CATH-2 analogs *in vitro*^18^.

Despite a different structural conformation, D-analogs of HDPs have been shown to have similar immunomodulatory effects to native L-peptides on several occasions^29^,^30^, possibly by activating signaling cascades through a receptor-independent effect on the host cell membrane. The concept of HDP analogs as immune modulators that are able to protect against infection was conclusively shown for the innate defense regulator peptide IDR-1^31^. In addition, *in ovo* injection of immunostimulatory CpG-ODN showed protective effects against multiple bacterial pathogens again showing that immunomodulation, even at this early age, can be a powerful prophylactic tool^23^,^32^. The challenges in these studies (*E. coli* and *Salmonella*) were given at 1 or 2 days post hatch. In the study reported here, we show that D-CATH-2 shows protective action at least up until 7 days post hatch, for broiler chickens a significant part of their lifespan.

In the case of mortality, morbidity as well as bacteriological parameters, *in ovo* administration of D-CATH-2 was more efficient at protecting the animals compared to i.m. administration after hatch, even though D-CATH-2 concentration was lower for *in ovo* administration. Similarly, protection against Marek’s disease improved when chicks were vaccinated *in ovo* compared to vaccination at the day of hatch as the earlier exposure to antigen led to higher antibody titers by the time of challenge^33^,^34^. The earlier exposure might also be a factor in the success of *in ovo* administration of D-CATH-2 in this study as *in ovo* administered peptide could affect the maturation of the immune system or the colonization of mucosal surfaces by microbiota directly after hatch, a window of opportunity which i.m. administration after hatch might miss. Alternatively, *in ovo* administered D-CATH-2 can lead to better protection of mucosal surfaces compared to i.m. injection which will generate a more systemic response. Immunostimulatory CpG-ODN was also reported to protect against bacterial infections both after *in ovo* and post hatch i.m. administration; however, the two modes of administration were not compared in the same study here^23^,^32^.

From vaccine research it is clear that the administration route of vaccines not only affects the magnitude of antibody response but also the type of immunity^35^. In our study IgG levels against *E. coli* were increased by both peptide treatments while IgM levels were significantly lower in *in ovo* treated animals compared to chicks that received D-CATH-2 i.m. As *in ovo* administered D-CATH-2 was shown to reach the bursa, the dedicated organ for B cell development in birds, a direct influence of the peptide on B cells is a possibility. The influence of HDPs on the adaptive immune system and B cells in particular has not been investigated extensively and results are somewhat contradictory. Serum IgG levels after vaccination of mice with ovalbumin were found to increase if the mouse cathelicidin CRAMP was co-administered^36^. A similar effect was reported for chicken CATH-1 injection in mice^37^. However, an enhanced antibody response to TNP-ovalbumin was also observed for CRAMP knockout mice^38^. Clearly, further investigation is needed to determine whether or not D-CATH-2 directly influences antibody production or if the differences in IgM levels are an indirect result of the partial protection.

Leukocyte numbers in blood were analyzed to obtain insight into the mechanisms behind the protective effect of D-CATH-2 and effects on multiple cell populations were observed. In the untreated infected group decreased lymphocyte numbers were observed, but no effect on total leukocytes and heterophils was seen, similar to the blood picture after *E. coli* challenge as described by Iseri *et al.*^39^ and indicative of the immunosuppression which has been ascribed to *E. coli* infections in chickens^40^. *In ovo* D-CATH-2 treatment caused an increase in total leukocyte numbers and both D-CATH-2 treatments counteracted infection-induced lymphopenia and increased heterophil numbers. Surprisingly however, very little effect was seen on monocyte numbers by both infection and treatment. The results in the D-CATH-2 treatment groups could partly be attributed to the decreased severity and thus reduced immunosuppression of infection in these animals although some differences, such as the increased leukocyte numbers point to an independent, treatment-related effect. CATH-2 and many other HDPs are known to induce chemokine expression in immune cells^18^,^37^. Chemotaxis and increased leukocyte recruitment to the site of infections were shown to be the main mechanism behind the anti-infective effect of the synthetic cationic peptide IDR-1002^41^. A similar importance might very well be attributed to increased leukocyte numbers in the D-CATH-2 treated animals. Alternatively, D-CATH-2 might affect leukocyte production in bone marrow, either already in the native animals or only in response to infection. This would be in line with a recent study showing an increase in leukocytes in zebrafish embryos treated with D-CATH-2^42^. Though cathelicidins have been shown to influence the differentiation of multiple types of leukocytes, influence on production of these cells has not been described as such^43^,^44^. The human cathelicidin LL-37 was however shown to positively influence recovery of white blood cell and platelet counts after irradiation for bone marrow transplantation^45^.

In conclusion, we have shown that the HDP analog D-CATH-2 is able to partially protect young broiler chickens against *E. coli* infection and that *in ovo* injection of D-CATH-2 is more successful compared to post hatch administration. Results of leukocyte counts and antibody measurements give a first insight into the possible mechanisms involved in this protection. D-CATH-2 seems able to modulate the chicken’s immune defenses, without causing a potentially harmful excessive inflammatory response. The results presented here are an important step in the search for alternatives to antibiotics and show that *in ovo* injection of HDP analogs can be a feasible means of infection control in the poultry sector.

## Material and Methods

### Synthesis of cathelicidin peptides

D-CATH-2 (amino acid sequence: RFGRFLRKIRRFRPKVTITIQGSARF-NH_2_) and N-terminally FITC and TAMRA labeled peptides were generated by solid-phase synthesis using Fmoc-chemistry and purified to >95% purity by RP-HPLC by CPC Scientific Inc. (Sunnyvale, USA), and the Academic Center for Dentistry (ACTA, Amsterdam, The Netherlands), respectively.

### Bacterial culture

*E. coli* strain 506 (O78, K80), originally isolated from a commercial broiler with colibacillosis^46^ was prepared by plating one frozen bead of *E. coli* 506 stock on sheep blood agar. The next day a single colony was transferred to 0.1% glucose broth (0.5% Lab Lemco powder (Oxoid, Basingstoke, UK), 1% bacteriological peptone (Oxoid), 0.5% NaCl) Overnight culture was diluted to a concentration of 10^7^ colony forming units (CFU)/ml with phosphate buffered saline (PBS) and kept on ice until inoculation. Bacterial concentration was verified by plating the diluted culture on sheep blood agar plates and performing a colony count.

### Animals

All experiments were conducted in accordance with protocols approved by the Dutch experimental animal committee (DEC). Eighteen-day-incubated Ross 308 broiler eggs were obtained from a commercial hatchery (Lagerwey, Lunteren, The Netherlands). Chickens were housed in negative pressure HEPA isolators with a wire floor. Isolators were ventilated at a rate of 40 m^3^/h and temperature was gradually decreased from 35 °C at day of hatch to 20 °C at 11 days of age. Light regime was 23 hours of light/1 hour of darkness and from 4 days of age onward isolators were illuminated with red light to prevent cannibalism. Chickens were fed a commercial broiler diet without antibiotics or coccidostats and were given access to food and water *ad libitum.*

### *In vivo* experiment I: localization of peptides

Three days before hatch, at 18 days of embryonic development, labeled peptides (FITC-D-CATH-2 or TAMRA-L-CATH-2) were administered *in ovo* (n = 3/group). Peptides were dissolved in PBS (1.48 mM NaH_2_PO_4_.H_2_0, 8.06 mM Na_2_HPO_4_, 20 mM NaCl, pH 7.27) to a concentration of 4.4 mg/ml to which cholesterol (5% v/v, 2 mg/ml in ethanol absolute) was added. Per egg, 100 μl mixture corresponding to 20 mg/kg bodyweight (estimated embryo weight = 22 g) was injected manually using a 1 inch, 21G needle. After 24 hours incubation embryos were euthanized by cervical dislocation and collected organs were snap-frozen in liquid nitrogen and stored at −20 °C until further analysis.

### Fluorescence microscopy and immunohistochemistry of labeled peptides

Cryostat sections (8–10 μm) from embryonic tissues were mounted on Superfrost Ultra Plus slides (Thermo Scientific, Waltham, USA), air-dried, fixed with cold acetone for 10 minutes, stained with DAPI and mounted with FluorSave (Calbiochem, San Diego, USA). Macrophages in lung sections were visualized by immunofluorescence using a FITC labeled anti-chicken monocyte/macrophage antibody (KUL-01, SouthernBiotech, Birmingham, USA). To this end, sections were blocked with 2.5% bovine serum albumin (BSA) for 1 hour, subsequently incubated with KUL-01 antibody (1:200) for 1 hour and counterstained with DAPI. To confirm fluorescent microscopy findings, sections were stained with antibodies against CATH-2^28^, FITC (Invitrogen, Waltham, USA) and TAMRA (Molecular Probes, Eugene, USA). Immunohistochemistry was performed as described by Van Dijk *et al.*^28^.

### *In vivo* experiment II: *E. coli* challenge

Chickens were divided into four treatment groups of 89 animals: 1) no challenge, no D-CATH-2 treatment, 2) *E. coli* challenge, no D-CATH-2 treatment (buffer), 3) *E. coli* challenge, *in ovo* D-CATH-2 treatment, 4) *E. coli* challenge, i.m. D-CATH-2 treatment. D-CATH-2 was appropriately diluted (0.22 mg/ml = 1 mg/kg bodyweight) and injected *in ovo* as described above (*In vivo* experiment I). After hatch (d0) and at 4 days of age, chickens were injected in the thigh muscle with either D-CATH-2 (final administration of 20 mg/kg bodyweight and 2.5 mg/kg bodyweight respectively) or buffer in a 100 μl volume. At 7 days of age, chickens were intratracheally inoculated with 0.3 ml of 10^7 ^CFU/ml of *E. coli* 506 or buffer using a trachea canule. Mortality was recorded daily in the following week. At 2 and 7 days post infection, animals were chosen at random for sample collection (n = 13 and n = 41 animals per group, respectively). Blood samples were taken from the wing vein. Animals were euthanized by electrocution followed by bleeding. Organ samples collected for histology and qPCR were placed in 4% formaldehyde solution or snap-frozen in liquid nitrogen respectively.

### Postmortem analysis

Colibacillosis lesions in surviving birds were scored macroscopically at 7 days p.i. in the left and right thoracic air sac, liver and pericardium, as described by Van Eck and Goren^46^. Briefly, lesion scores can range between 0 (healthy) and 3 (heavily diseased) per organ. The Mean Lesion Score (MLS) was calculated as the sum of scores per bird.

### Bacterial numbers in air sacs and blood

Thoracic air sacs of each bird were swabbed immediately after postmortem examination using transport swabs with Amies medium (Oxoid, Basingstoke, UK) and plated on MacConkey/Sheep Blood agar biplates (bioTRADING, Mijdrecht, The Netherlands) within 16 hours. Blood (100 μl) was plated on MacConkey/Sheep Blood agar biplates immediately after collection. Bacterial growth was evaluated after overnight incubation at 37 °C.

### Leukocyte counts

Blood smears were prepared from samples taken at 2 days p.i. (n = 13/group), air dried, fixed in methanol for 10 minutes and stained with May-Grünwald & Giemsa (Merck, Darmstadt, Germany). Per slide 100 leukocytes were counted and differentiated blindly by trained individuals.

### Antibody measurements (ELISA)

Cell culture plates (96-well) were coated with *E. coli* 506 lysate, blocked with 5% BSA and incubated with chicken serum samples for 1.5 hours. Horse Radish Peroxidase (HRP) labeled anti-IgG or anti-IgM was used as a secondary antibody (1:2000, AbD Serotec, Kidlington, UK). Bound antibody was visualized with the TMB Substrate Reagent Set (BD Biosciences, San Jose, USA). Absorbance was measured at 450 nm in a microplate reader. Relative antibody concentrations were calculated based on a serial dilution curve of pooled serum samples.

### Histological analysis

Lung samples (n = 11 − 13/group) were fixed in 4% formaldehyde for 24 hours and paraffin-embedded. Paraffin sections of 5 μm were mounted on glass slides, deparaffinized and rehydrated for *E. coli* immunohistochemistry and Terminal Deoxynucleotidyl Transferase dUTP Nick End Labeling (TUNEL) stain. *E. coli* immunohistochemistry was performed using antiserum raised in rabbits against killed *E. coli* 506 27. To this end, lung sections were incubated for 30 min with 1% H_2_O_2_ in methanol to block endogenous peroxidase activity and subsequently blocked with 10% normal goat serum and 2.5% BSA for 1 hour. After staining with *E. coli* antiserum (1:1000) for 1 hour, sections were incubated with the HRP labeled anti-rabbit polymer from the EnVision+ system (Dako, Glosstrup, Denmark). HRP staining was visualized by incubating the sections with diaminobenzidine (DAB) for 5–10 minutes. Sections were counterstained with haematoxylin, dehydrated and mounted with Pertex. TUNEL staining was performed to analyze the number of apoptotic cells in lungs. The assay was carried out as described by Horn *et al.*^47^ with modifications. Briefly, sections were incubated with 20 μg/ml proteinase K for 15 min. Subsequently, endogenous peroxidase activity was blocked using 2% H_2_O_2_ in TBS. Sections were then blocked using 20% FCS and 1% BSA in TBS for 1 hour followed by incubation in Terminal Deoxynucleotidyl Transferase (TdT) buffer (0.2 M potassium cacodylate, 1 mM cobalt chloride, 25 mM Tris and 0.1% Triton X-100) for 15 min. Nick-end labeling was performed by incubating the slides with TdT (20 U/μl, Thermo Scientific, Waltham, USA) and biotinylated dUTP (1 mM, Roche, Mannheim, Germany) in TdT buffer for 1 hour at 37 °C. The labeling reaction was terminated by washing sections in TB buffer (0.3 M natrium chloride, 30 mM trisodium citrate). To visualize TUNEL labeled cells, sections were incubated with Avidin-Biotin complex (VectaStain Elite ABC Kit, Vector Laboratories, Burlingame, USA) for 30 min and subsequently with diaminobenzidine for 5 min. Sections were counterstained with haematoxylin, dehydrated and mounted with Pertex. TUNEL stained cells were analyzed using an Image J based plugin counting ten fields for each sample.

### Real time qPCR

RNA was isolated using MagNA Lyser Green Beads and the High Pure RNA Tissue Kit (Roche, Mannheim, Germany) according to the manufacturer’s instructions. RNA purity was assessed by NanoDrop and integrity by running the RNA samples on an agarose gel. Isolated RNA was DNase treated (DNase I recombinant, Roche, Mannheim, Germany) and checked for residual DNA contamination by β-actin PCR on RNA. RNA (100 ng) was converted into cDNA in a 10 μl reaction using the iScript cDNA Synthesis Kit (BioRad, Hercules, USA) according to the kit’s instructions. Real time qPCR was performed in duplicate for each biological sample (using 5 μl of 10× diluted cDNA/reaction) using iQ Supermix (BioRad, Hercules, USA) in a final volume of 12.5 μl/reaction. Standard curves were generated by serial dilution of pooled cDNA from all experimental samples and were incorporated in all qPCR runs. Primers/probes sequences, qPCR conditions and R^2^ and efficiency values are depicted in Supplementary Table S1. Fold changes of the target genes were calculated using the qBase+ analysis software (Biogazelle, Zwijnbeke, Belgium) and normalized against GAPDH and 28S expression^48^.

### Statistical analysis

Statistical analysis was performed using SPSS 22 software (IBM, Armonk, USA). Differences between treatment groups were evaluated using a one-way analysis of variance (ANOVA) or non-parametric Kruskal-Wallis test when data were non-normally distributed. A Chi-Square test was used to analyze differences in numbers of animals with an MLS > 1 or positive for *E. coli* in blood and air sacs. Kaplan-Meier analysis was used to compare survival between groups. Differences were considered statistically significant if p < 0.05.

## Additional Information

**How to cite this article**: Cuperus, T. *et al.* Protective effect of *in ovo* treatment with the chicken cathelicidin analog D-CATH-2 against avian pathogenic *E. coli. Sci. Rep.* **6**, 26622; doi: 10.1038/srep26622 (2016).

## Supplementary Material

Supplementary Information

## Figures and Tables

**Table 1:** Number of animals with colibacillosis (MLS > 1) at 7 days post *E. coli* challenge.

Group	Colibacillosis positive
uninfected	0% (0/41)
infected	66% (27/41)
+ *in ovo* D-CATH-2	32% (13/41)**
+ i.m. D-CATH-2	41% (17/41)*

Data were analyzed by a Chi-Square test. *p < 0.05, **p < 0.01, compared to infected.

**Table 2:** Number of animals with positive air sac swab and blood cultures (CFU > 5/plate) at 7 days post *E. coli* challenge.

Group	Air sacs	Blood
uninfected	13% (5/41)	0% (0/41)
infected	49% (20/41)	22% (9/41)
+ *in ovo* D-CATH-2	17% (7/41)**	2% (1/41)*
+ i.m. D-CATH-2	78% (32/41)*	15% (6/41)

Data were analyzed by a Chi-Square test. *p < 0.05, **p < 0.01, compared to infected.
