# Supplementary material for: Protective effect of in ovo treatment with the chicken cathelicidin analog D-CATH-2 against avian pathogenic E. coli
Source: Sci Rep. 2016 May 27;6:26622. doi: 10.1038/srep26622 (PMC4882517; doi:10.1038/srep26622)
Supplement: Supplementary Information [file srep26622-s1.pdf]

Supplementary information for:

Protective effect of *in ovo* treatment with the chicken cathelicidin analog D-CATH-2 against avian pathogenic *E. coli*

Tryntsje Cuperus[1], Albert van Dijk[1], Mieke G.R. Matthijs[2], Edwin J.A. Veldhuizen[1], Henk P. Haagsman[1*]

[1]Division of Molecular Host Defence, Department of Infectious Diseases & Immunology, Faculty of Veterinary Medicine, Utrecht University, Utrecht, The Netherlands; [2]Division of Poultry Health, Department of Farm Animal Health, Faculty of Veterinary Medicine, Utrecht University, Utrecht, The Netherlands

* Address correspondence to Henk P. Haagsman, h.p.haagsman@uu.nl

T.C. and A.D. contributed equally to this work.

Supplementary table S1: Real Time qPCR primers, probes, R^2 and efficiency values.

Gene		Sequence (5'-3')	R^2	Efficiency (%)
GAPDH	Forward	GCCGTCCTCTCTGGCAAAG	0.967	94
	Reverse	TGTAAACCATGTAGTTCAGATCGATGA		
	Probe	AGTGGTGGCCATCAATGATCCC		
28S	Forward	GGCGAAGCCAGAGGAAACT	0.977	108
	Reverse	GACGACCGATTTGCACGTC		
	Probe	AGGACCGCTACGGACCTCCACCA		
AvBD9	Forward	GCTGTTCTCTTCTTCCTC	0.968	84
	Reverse	CATGCAACAAAAGAGCAG		
	Probe	TGCTCCAGCTTACAGCCAAGA		
CATH-2	Forward	TACAACCAACGGCCTGAG	0.935	101
	Reverse	CGAGCAGTCCCTGATGAC		
	Probe	CTCCTTGAAGTCGCAGTCG		
IL-1β	Forward	GCTCTACATGTCGTGTGTGATGAG	0.907	100
	Reverse	TGTCGATGTCCCGCATGA		
	Probe	CCACACTGCAGCTGGAGGAAGCC		
IL-6	Forward	GTCGAGTCTCTGTGCTAC	0.840	98
	Reverse	GTCTGGGATGACCACTTC		
	Probe	ACGATCCGGCAGATGGTGA		
IL-8	Forward	GCCCTCCTCCTGGTT TCA	0.920	103
(chCXCLi2)	Reverse	CGCAGCTCATTCCCCATCT		
	Probe	TGCTCTGTCGCAAGGTAGGACGCTG		
iNOS	Forward	CCAAGATCCAGAAATATTTGA	0.870	99
	Reverse	ACTCCTTAAACCACTCATA		
	Probe	CGCCAGAGATTATCCTTGAAGTGC		
MCP-3	Forward	CTGGTGCTTCTCCTATGTTCAAC	0.975	136
(chCCLi7)	Reverse	ACACATATCTCCCTCCCTTTGTTG		
	Probe	CTACTCCACTCCCATCCACCAGCATTG		
RANTES	Forward	CCCTCTCCATCCTCCTGGTT	0.847	93
(chCCLi4)	Reverse	TATCAGCCCCAAACGGAGAT		
	Probe	CCGCCCTCTTCCCTCAAGCCTC		

Probes were labeled with 6-carboxyfluorescein (FAM) at the 5' side and Black Hole Quencher-1 at the 3' side, R^2 and efficiency values are average values over multiple qPCR runs. PCR program used: initial denaturation for 5 min at 95°C, followed by 40 cycles of 95°C for 20 seconds, 60°C for 30 seconds and 72°C for 40 seconds.

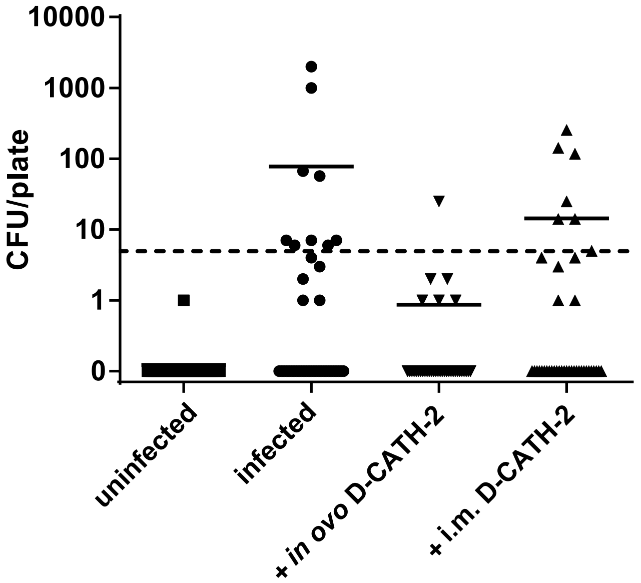

Supplementary Figure S1: *E. coli* counts in blood at 7 days post *E. coli* challenge. Blood (100 µl) was plated on MacConkey/Sheep Blood agar plates and colonies were counted after overnight incubation. Dotted line indicates CFU=5, above which animals are considered *E. coli* positive (Table 2). Data were analyzed by the non-parametric Kruskal-Wallis method.

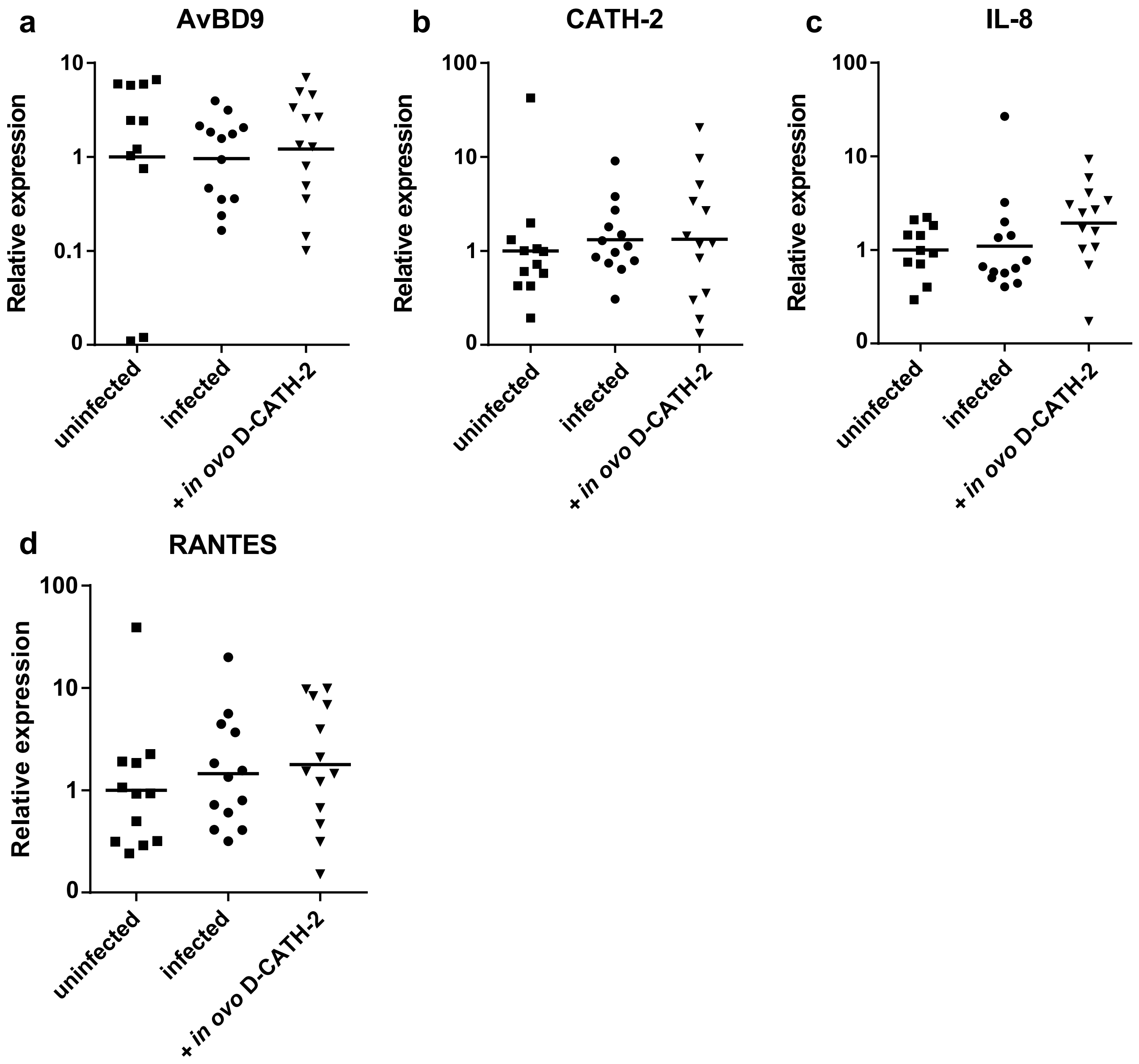

Supplementary Figure S2: Immune related gene expression in lung measured at 2 days post *E. coli* challenge by qPCR. Expression was calculated relative to the uninfected group. Geometric means are displayed. a. AvBD9, b. CATH-2, c. IL-8 and d. RANTES. Data were analyzed by one-way ANOVA with a Tukey post-hoc test.

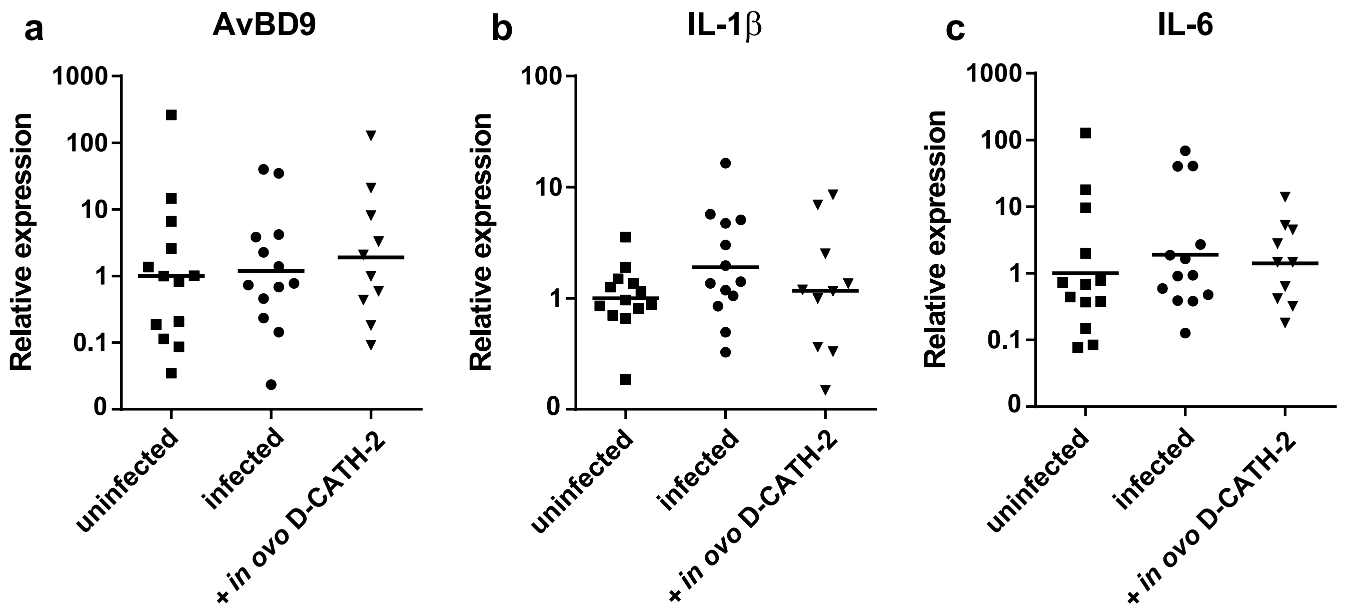

Supplementary Figure S3: Immune related gene expression in spleen measured at 2 days post *E. coli* challenge by qPCR. Expression was calculated relative to the uninfected group. Geometric means are displayed. a. AvBD9, b. IL-1β, c. IL-6. Data were analyzed by one-way ANOVA with a Tukey post-hoc test.
